# A core-shell molybdenum nanoparticles entrapped *f*-MWCNT*s* hybrid nanostructured material based non-enzymatic biosensor for electrochemical detection of dopamine neurotransmitter in biological samples

**DOI:** 10.1038/s41598-019-48999-0

**Published:** 2019-09-10

**Authors:** Murugan Keerthi, Gopal Boopathy, Shen-Ming Chen, Tse-Wei Chen, Bih-Show Lou

**Affiliations:** 1 Department of Chemical Engineering and Biotechnology, National Taipei University of Technology, Taipei, 106 Taiwan; 2 Chemistry Division, Center for General Education, Chang Gung University, Taoyuan, 333 Taiwan; 3 Department of Nuclear Medicine and Molecular Imaging Center, Chang Gung Memorial Hospital, Taoyuan, 333 Taiwan

**Keywords:** Electrocatalysis, Electrocatalysis, Chemical engineering, Chemical engineering

## Abstract

Dopamine (DA) is a critical neurotransmitter and has been known to be liable for several neurological diseases. Hence, its sensitive and selective detection is essential for the early diagnosis of diseases related to abnormal levels of DA. In this study, we reported novel molybdenum nanoparticles self-supported functionalized multiwalled carbon nanotubes (Mo NPs@*f*-MWCNTs) based core-shell hybrid nanomaterial with an average diameter of 40–45 nm was found to be the best for electrochemical DA detection. The Mo NPs@*f*-MWCNTs hybrid material possesses tremendous superiority in the DA sensing is mainly due to the large surface area and numerous electroactive sites. The morphological and structural characteristics of the as-synthesized hybrid nanomaterial were examined by XRD, Raman, FE-SEM, HR-TEM, EDX. The electrochemical characteristics and catalytic behavior of the as-prepared Mo NPs@*f*-MWCNTs modified screen-printed carbon electrode for the determination of DA were systematically investigated via electrochemical impedance spectroscopy, cyclic voltammetry, and amperometry. The results demonstrate that the developed DA biosensor exhibit a low detection limit of 1.26 nM, excellent linear response of 0.01 µM to 1609 µM with good sensitivity of 4.925 µA µM^−1^ cm^−2^. We proposed outstanding appreciable stability sensor was expressed to the real-time detection of DA in the real sample analysis of rat brain, human blood serum, and DA hydrochloride injection.

## Introduction

Dopamine (DA, 3,4-dihydroxyphenylalanine) is a catecholamine neurotransmitter, which plays an important biological role in human metabolism, cardiovascular, central nervous, renal, and hormonal systems^1^. Since DA is critical for signal transmissions to the brain^2–4^. The normal concentration of DA is between 10 and 1000 nM L^−1^ ^5–7^, abnormal and inadequate level of DA can lead to many neurological disorders such as Parkinson’s disease (PD), schizophrenia, hypertension, and attention deficit hyperactivity disorder (ADHD)^1,5,8,9^. Therefore, the trace level detection of DA in a biological sample has been much important in the analytical and biomedical application for the early diagnosis and prevention^10,11^. It is necessary to design DA biosensor with excellent characteristics such as high sensitivity and excellent selectivity. Recently several analytical methods have been proposed for the DA detection such as high-performance liquid chromatography (HPLC)^12^, chemiluminescence^13^, fluorescence^14^, capillary electrophoresis^15^, mass spectrometry, calorimetry^16^, and surface-enhanced Raman scattering spectroscopy^17^. Although, these methods associated with some drawbacks such as require complicated pretreatment, time-consuming, expensive instruments, and consume large quantities of chemical, solvents, and reagents which might possibly cause environmental pollution^18,19^. Expectedly, the characteristics of the electrochemical behavior of DA, make it easily detectable by facile electrochemical method. To date, electrochemical techniques have been extensively explored for the detection of DA owing to their remarkable properties such as fast detection, facile operation mode, accessibility, cost-effectiveness, simplicity, and eco-friendly^4,20,21^. However, the electrochemical detection of DA in a biological fluid using bare electrode is often unsuccessful due to interfering molecules such as an Ascorbic acid (AA) and Uric acid (UA) coexisting with DA detection because of its similar oxidation potential^22^. As a result, the accuracy of its determination is remarkably low, so it is impossible to avoid their presence in real samples. The basal concentration of DA is very low (0.01–1 µM), while the concentration of AA and UA is much higher than that of DA (from 100 to 1000 times higher)^23,24^. Nowadays, the variously modified electrodes have been developed for the sensitive and selective DA detection in the routine analysis without the problem of some potential interferents. Though, these modified electrodes are unsuitable for the specific detection of DA due to its poor selectivity, sensing in higher oxidation potential and fouling to the signal response. To overcome this issue, the modified electrode with those requirements that can be employed by exploiting the hybrid nanomaterials.

So far, numerous hybrid nanomaterials have been fabricated for the electrochemical biosensor application. Especially MWCNTs, the unique carbon-based material consists of curled cylindrical tubes, have been widely used in fabricating electrochemical sensors due to their overwhelming advantages such as the high intrinsic carrier mobility, large surface/volume ratio, good flexibility, and conductivity^25–30^. Nevertheless, pristine MWCNT alone is unable to produce hydrophilicity. Functionalization of MWCNTs by acid condensation method using concentrated acids such as sulfuric acid (H_2_SO_4_) and nitric acid (HNO_3_) imparts higher solubility and allowing them as a catalytic active material for the electrochemical applications^31^. The chemical oxidation of MWCNTs introduces more oxygen functional groups on their surface, which improve their hydrophilicity, chemical reactivity and intended to enhance dispersity of MWCNT in an aqueous solution^32^. The acid mixture has the ability to break the tubes of MWCNTs resulting in an increase in their electrical conductivity and corrosion resistance. Furthermore, due to the presence of abundant functional groups and delocalized π bonding, *f*-MWCNTs are suitable matrix to anchor metal/metal oxide nanoparticle^33^. In recent days, *f*-MWCNT supported metal nanoparticles nanocomposite widely studied in the field of electrochemistry to improve the electrocatalytic properties^34^. Among these metal nanoparticles, Molybdenum based materials act as a noble metal-free electrocatalyst which played a prominent role in hydrogen production even with lower over potential^35^. Molybdenum-based nanomaterials have increased much research attention in recent times because of their ultrahigh specific surface area and unique optical, electronic, catalytic, and mechanical properties^36–38^. To prepare Mo nanoparticles, many advanced synthesis strategies have been utilized, such as a molten salt technique^39^, carbothermal reduction^40^, hydrogen reduction^41^, microwave assisted combustive reduction and electro reduction^42^. However, these synthetic techniques are owing to the complicated preparation method, high reduction temperature and costly equipment ultimately resulted in casing research on facile and low-temperature synthetic method for the production of Mo nanoparticles. In this paper, Solution combustion synthesis (SCS) is adopted to prepare Mo nanoparticle. The SCS method is a simple and low production cost and energy efficient method^43^, which is based on a redox reaction between fuel and oxidant containing metal cations. Usually, metal precursors themselves act as an oxidant and fuel is an organic reagent for examples citric acid, ethylene glycol, and urea, etc. has the ability to form a complex with the metal cations^44^.

Hence, in this study, a core-shell hybrid nanomaterial of Mo NPs@*f*-MWCNTs was successfully synthesized by acid condensation method and used to detect DA electrochemically for the first time. Where the negatively charged oxygen functional groups on the MWCNT serve as anchor sites for interaction of positively charged Mo NPs on the surface and cover the nanotubes through the robust electrostatic interaction. Definitively, the as-prepared Mo NPs@*f*-MWCNTs was employed as the excellent electrocatalytic material for the detection of DA in both buffer and biological samples.

## Experimental

### Chemicals and apparatus

Multiwalled carbon nanotubes (MWCNTs) (95%, O.D × I.D × length = 7–15 × 3–6 × 0.5–200 µm), Dopamine, Hexaammonium molybdate (NH_4_)_6_Mo_7_O_24_⋅4H_2_O, ammonium nitrate (NH_4_NO_3_), glycine and all the required chemicals were purchased from Sigma-Aldrich. Dopamine hydrochloride injection was purchased from the local medical hospital. Rat brain sample was carried out in accordance with local laws and approved by Chang Gung University, IACUC Approval No. CGU105-079. The human serum sample used in this study was obtained with written informed consent from all subjects. Human serum was acquired from a healthier volunteer in Chang Gung Memorial Hospital and this study was approved by the institutional review board of Chang Gung Memorial Hospital, IRB No. 201601498B0. All experiments involving the human samples were performed in accordance with relevant guidelines and regulations of Chang Gung Memorial Hospital. The electrolyte of phosphate buffer (PB, 0.1 M) was prepared by mixing Na_2_HPO_4_ and NaH_2_PO_4_. H_2_SO_4_ and NaOH were used for optimizing the pH of the solution. The morphological characterization was studied using Hitachi S-3000 H (FE-SEM) and (HR-TEM) H-7600, Hitachi, (Japan). The elemental percentage and composition of the materials were investigated with the help of Energy-dispersive X-ray (EDX) HORIBA EMAX X-ACT (Sensor + 24 V = 16 W, resolution at 5.9 keV). Crystalline structure confirmed via Powder X-ray diffraction (XRD) XPERT-PRO (PAN analytical B.V. The Netherlands) diffractometer with Cu Kα radiation (k = 1.54 Å). Raman spectra were performed using Micro-Raman spectrometer. The electrochemical measurements were performed by cyclic voltammetry (CV) CHI 205 C and Amperometric (i-t) measurements were obtained with an analytical rotator with a working area of 0.21 cm^2^ of rotating ring disc electrode (RRDE), platinum wire as the counter electrode, and Ag/AgCl (saturated KCl) as the reference electrode. All the experiments were performed triplicate.

### Synthesis of Mo nanoparticles

The Mo NPs were prepared according to the earlier reported procedure with minor modification^37^. The SCS method was utilized in the preparation of the Mo precursor. First, 0.01 M (NH_4_)_6_Mo_7_O_24_⋅4H_2_O, 0.2 M NH_4_NO_3_, and 0.1 M glycine were dissolved in 100 mL deionized water (DI). The mixer was stirred under magnetic stirrer until the homogeneous solution formed. The above dispersion was heated using a temperature controlled electric heating furnace. After several minutes, a black precipitate of MoO_2_ precursor was obtained. Mo NPs were synthesized from MoO_2_ by hydrogen reduction process in a tubular furnace. The MoO_2_ was heated up to 2 h under programmed heating rate (10 °C/min; 500 °C, 550 °C, 600 °C, 650 °C, and 700 °C). During the reaction, 100 mL/min of H_2_ (99.99%) was maintained during hydrogen reduction. The obtained Mo NPs was allowed to cool room temperature naturally.

### Synthesis of core-shell Mo NPs@*f*-MWCNTs hybrid nanocomposite

The Mo NPs@*f*-MWCNTs nanocomposite was prepared using an acid condensation method. In the typical synthesis procedure, 1:5 ratio of Mo nanoparticle and MWCNTs were taken in the round bottom flask and then (3:1) H_2_SO_4_ and HNO_3_ were added to the above mixer^32^. The solution then condensed at 24 h under magnetic stirring. The obtained Mo NPs@*f*-MWCNTs nanocomposite was centrifuged and washed several time with DI water and ethanol to remove the unreacted particles in the product. The *f*-MWCNTs were prepared without adding Mo NPs for control studies.

### Fabrication of Mo NPs@f-MWCNTs modified SPCE

**Figure 1:**
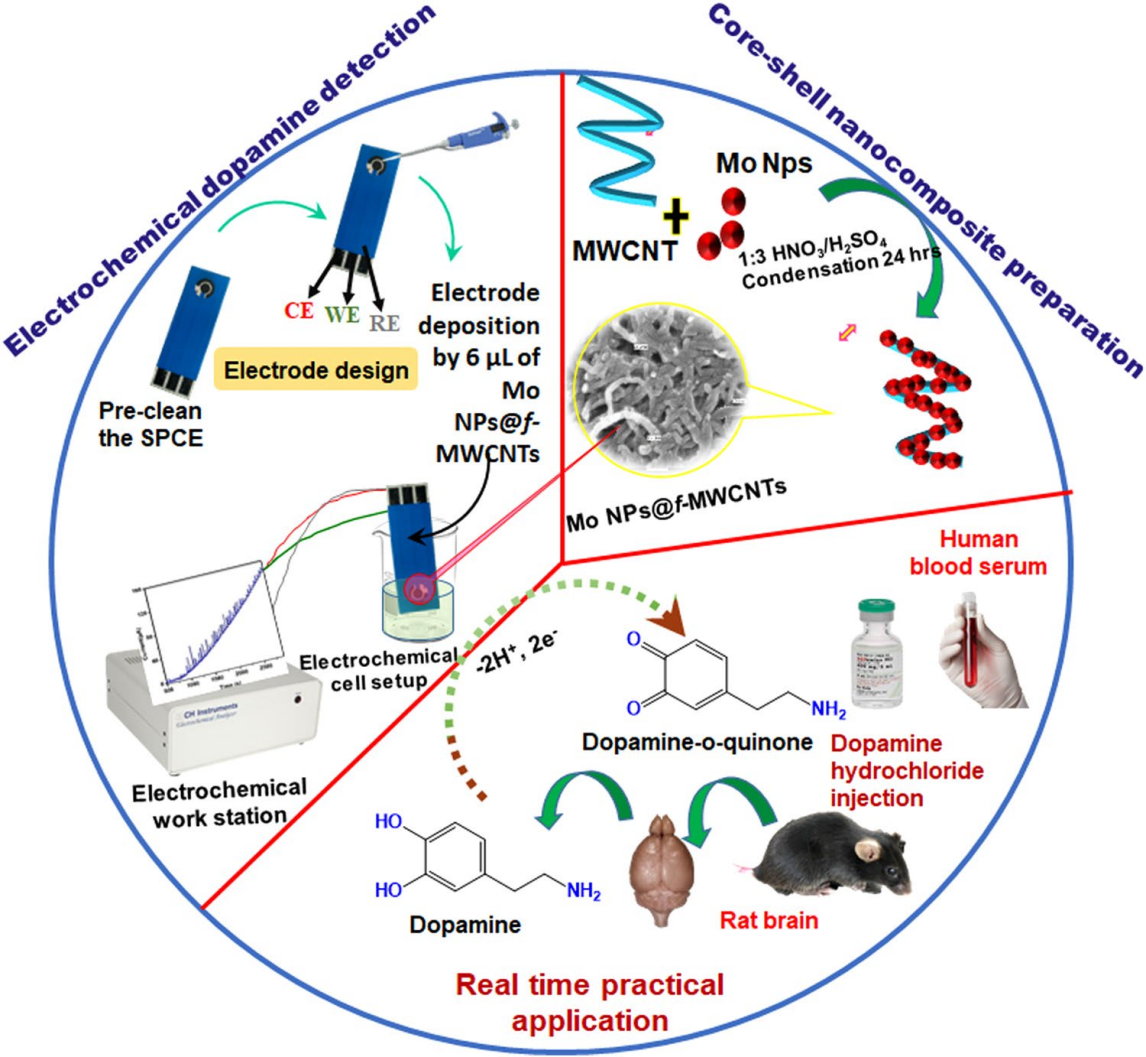
Schematic representation for the preparation of core-shell Mo NPs@*f*-MWCNTs hybrid nanocomposite and its electrochemical determination of neurotransmitter in biological samples.

The SPCE was modified by simple drop cast method. First, the working electrode (SPCE) surface was pre-cleaned by potential cycling between −1.0 V and 1.2 V in 0.1 M phosphate buffer (PB) (pH 7.0) for 5 cycles at a scan rate of 25 mV s^−1^. Typically, ~6 µL of dispersed Mo NPs@*f*-MWCNTs nanocomposite (optimized amount) was drop cast onto the SPCE electrode (Fig. 1). Then SPCE was allowed to dry in air at room temperature, followed by gently dipped with buffer solution for twice to remove the unbound material prior to further electrochemical measurements and a similar procedure was adhered to prepare control electrode (*f*-MWCNTs).

## Results and Discussion

### Structural characteristics of Mo NPs@*f*-MWCNTs nanocomposite

**Figure 2:**
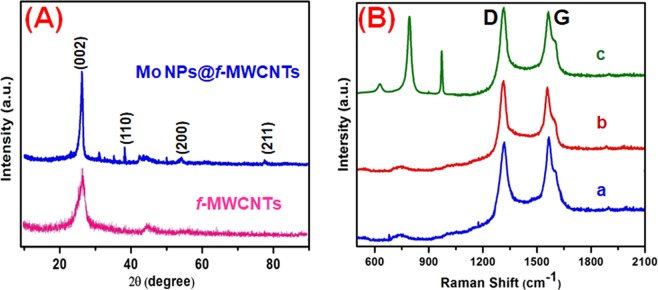
The XRD pattern (**A**) and Raman spectra (**B**) of pristine MWCNTs (a), *f*-MWCNTs (b), Mo NPs@*f*-MWCNTs nanocomposite (c).

Figure 2A displays the XRD patterns of *f*-MWCNTs and Mo NPs@*f*-MWCNTs nanocomposite. The diffraction pattern of *f*-MWCNTs featured with a characteristic peak at 2θ of 26.3° indexed to (002) that can be correlated to graphitic structure^32^. The XRD pattern of Mo NPs@*f*-MWCNTs featured with peaks at 27.2°, 40.1°, 54.2°, and 74.2° corresponding to the lattice planes of (002), (110), (200), and (211), respectively. The XRD pattern of Mo NPs@*f*-MWCNTs is consistent with the characteristic pattern of Mo nanoparticle (ICDD cards No. of 89-5023) as well as it included with the characteristic band of *f*-MWCNTs^37,39^.

Raman spectroscopy was conducted to confirm the degree of disorder in the MWCNTs structure during the composite formation. Figure 2B shows the Raman spectra of the pristine MWCNTs (a), *f*-MWCNTs (b), and Mo NPs@*f*-MWCNTs composites (c). The G-band at 1590 cm^−1^ is associated with the E_2_g plane in Stretching and vibration modes of the basal plane of graphite, which indicates that the existence of crystalline nature of graphitic carbon in the MWCNT samples. The peak at 1304 cm^−1^ (D-band) is assigned to the disordered carbon atoms of MWCNTs and defects. The intensities ratio between G-band (*I*_G_) and D-band (*I*_D_) is sensitive to chemical modification and its measure of defects in MWCNTs. Here, the value of *I*_D_/*I*_G_ for MWCNTs, *f*-MWCNTs, and Mo NPs@*f*-MWCNTs are found to be 0.93, 1.03, and 1.01. Expectedly, the *I*_D_/*I*_G_ value of *f*-MWCNTs was increased compared with pristine MWCNTs due to the introduction of plenty of defects during the functionalization process. However, there is no significant difference in the *I*_D_/*I*_G_ values of *f*-MWCNTs and Mo NPs@*f*-MWCNTs, which indicates no structural changes during the formation of the composite. The strong bands are obtained in the region of 600–1000 cm^−1^. The shape of the spectrum below 1000 cm^−1^ closely resembles that of the amorphous medium of molybdenum oxide or molybdenum hydroxide, as confirmed by previous reports^45,46^. the characteristic band of Molybdenum oxide due to electrostatic interaction of Mo nanoparticles with oxygen functional groups on the *f*-MWCNTs.

### Morphological characteristics of Mo NPs@*f*-MWCNTs nanocomposite

**Figure 3:**
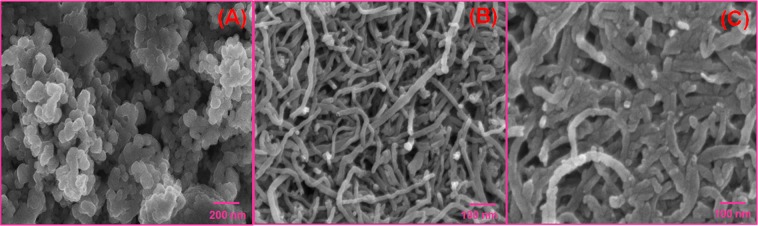
The FE-SEM images of Mo nanoparticles (**A**), *f*-MWCNTs and Mo NPs@*f*-MWCNTs Core-shell nanocomposite (**C**).

The morphology and nanostructure of the as-prepared Mo NPs, *f*-MWCNTs and Mo NPs@f-MWCNTs nanocomposite were investigated by FE-SEM. Figure 3A representing Mo nanoparticles, which seems to be a spherical shape. As shown in Fig. 3B, the size distribution of *f*-MWCNTs is relatively uniform with a smooth, dense, and typical tubular structure interlinking with each other. As a comparison, the Mo NPs@*f*-MWCNTs exhibits a rough surface and much wider than *f*-MWCNTs with a significant morphological change (Fig. 3C). The increment of tube size is due to Mo nanoparticles covered with *f*-MWCNTs through strong electrostatic interaction between negatively charged oxygen functional groups on the *f*-MWCNTs surface and positively charged Mo nanoparticles. The central cores of nanotubes still retained the tube-like structure. whereas, it can be clearly observed that the nanotubes (cores) are covered with Mo NPs (shells) indicating the successful synthesis of a core-shell hybrid structure of Mo NPs@*f*-MWCNTs.

**Figure 4:**
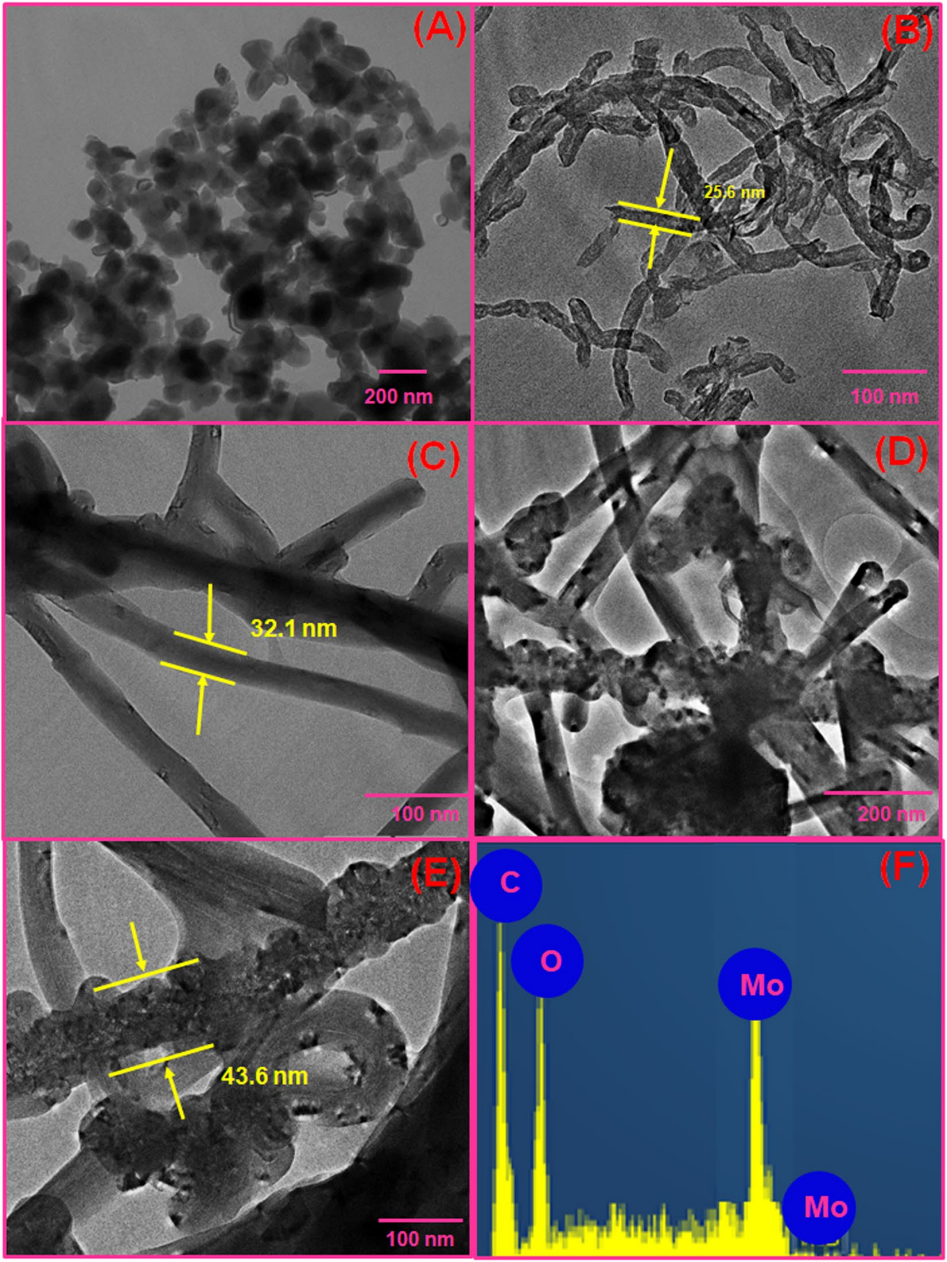
HR-TEM images of Mo nanoparticles (**A**), Pristine MWCNTs (**B**), *f*-MWCNTs (**C**), Mo NPs@*f*-MWCNTs (**D**,**E**) and EDAX spectrum of Mo NPs@*f*-MWCNTs nanocomposite (**F**).

The TEM analysis was conducted to compare the morphologies and to confirm the detailed core-shell structure of Mo NPs@*f*-MWCNTs. Figure 4A shows that Mo NPs has irregular spherical structure, which seems to be agglomerated. For the comparison, TEM image with the outer diameter of the pristine MWCNTs and *f*-MWCNTs tubes displayed in Fig. 4B,C, the outer diameter of the *f*-MWCNTs increased to ~32.1 nm which is thicker than that of pristine MWCNTs (~25.6 nm) due to the formation of a chemical layer on its structure. The chemical layer of carboxyl groups attached to the MWCNTs during the acid functionalization. The TEM images of Mo NPs@*f*-MWCNTs (Fig. 4D,E) clearly shows the Mo NPs (shell) entrapped the *f*-MWCNTs (core), which further increase the size. The average diameter of the single tube in Mo NPs@*f*-MWCNT composite is calculated to be ~ 43.6 nm. The chemical composition of Mo NPs@*f*-MWCNTs was also characterized by EDAX and shown in Fig. 3F. The presence of C, O, Mo with the elemental percentage of 56.24, 25.54 and 18.22 respectively, revealed that the formation of the expected nanocomposite.

### Electrocatalytic property of Mo NPs@*f*-MWCNTs modified SPCE

**Figure 5:**
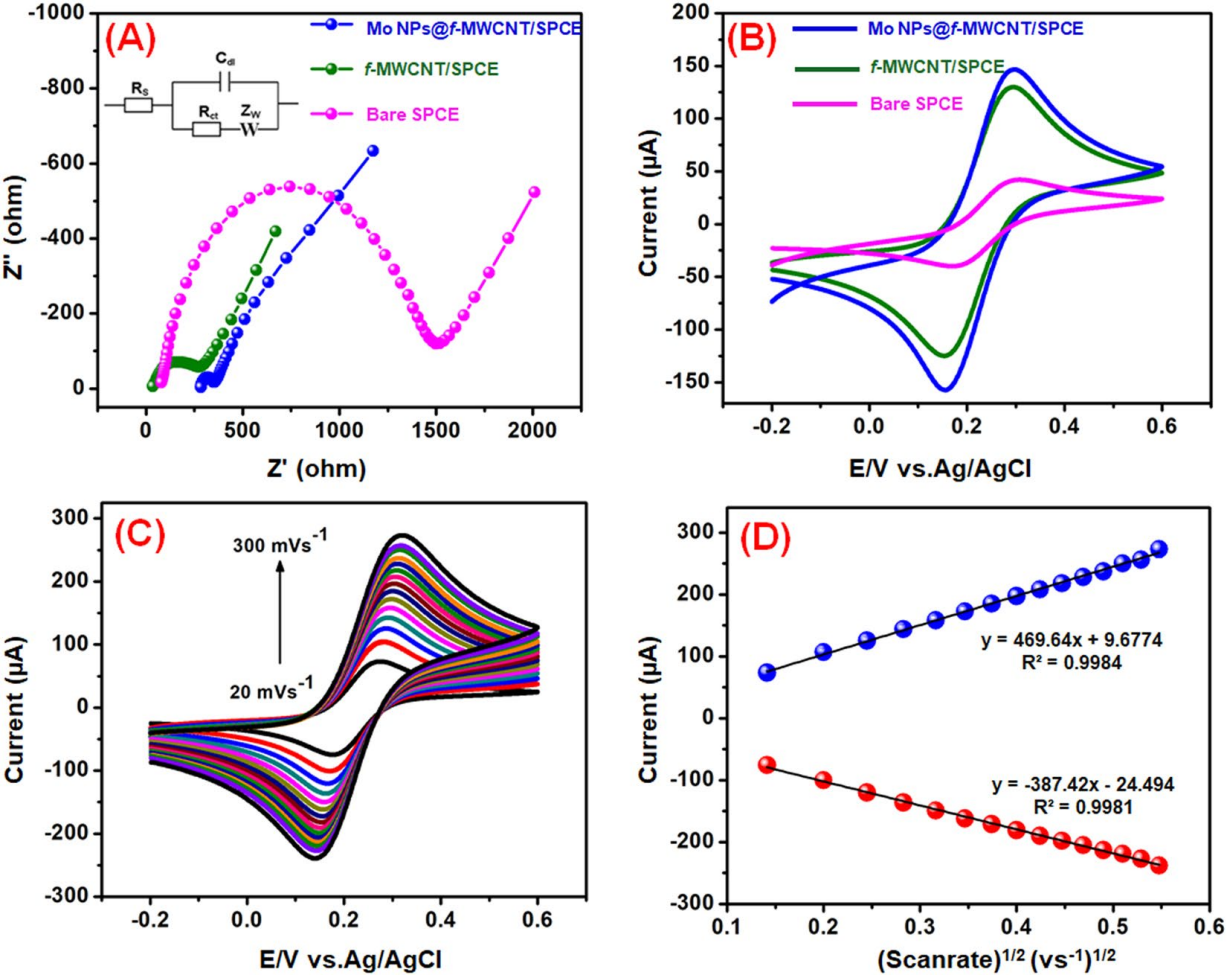
Electrochemical impedance spectra of bare SPCE, *f*-MWCNTs and Mo NPs@*f*-MWCNTs modified electrode (**A**). CVs of bare SPCE, *f*-MWCNTs and Mo NPs@*f*-MWCNTs modified electrode in 5 mM [Fe(CN)_6_]^3−/4−^ with 0.1 M KCl solution at 50 mV s^−1^ (**B**). CVs of the Mo NPs@*f*-MWCNTs/SPCE in 5 mM [Fe(CN)_6_]^3−/4−^ with 0.1 M KCl at scan rates of 20–300 mV s^−1^ (**C**). The linear plot for redox (*I*_pa_ and *I*_pc_) peak current vs. scan rate (D).

The electron transfer ability of the modified SPCE and bare SPCE studied by electrochemical impedance spectroscopy (EIS) in 5 mM [Fe(CN)_6_] ^−3/−4^ with 0.1 M KCl solution (Fig. 5A). The EIS data of bare SPCE, *f*-MWCNTs/SPCE, and Mo NPs@*f*-MWCNTs/SPCE were plotted using the Randles circuit model (Inset: Fig. 5A). Where, *R*_ct_, *R*_s_, *Z*_w_, and *C*_dl_ were portraying charge transfer resistance, electrolyte solution resistance, Warburg impedance, and double layer capacitance, respectively. From this EIS plot, it can be seen that all the modified electrode exhibited semicircles of different diameters which correspond to different *R*_ct_ value. The *R*_ct_ value obtained for bare SPCE, *f*-MWCNT/SPCE and Mo NPs@*f*-MWCNTs/SPCE was 236 Ω, 195 Ω, and 69 Ω respectively. Furthermore, the Mo NPs@*f*-MWCNTs/SPCE displays the very smaller semicircle (*R*_ct_ = 69 Ω) compared to *f*-MWCNT/SPCE and bare SPCE, which reveals that the composite modified electrode has superior electron transfer ability owing to its unique core-shell structure.

The electrocatalytic ability of bare SPCE, *f*-MWCNTs/SPCE, and Mo NPs@*f*-MWCNTs/SPCE were tested in cyclic voltammetry via using redox probe of 5 mM K_3_[Fe(CN)_6_]^3−/4−^ and 0.1 M KCl as supporting electrolyte (Fig. 5B). All the electrodes exhibited that the apparent redox peaks for the redox reaction of [Fe(CN)_6_]^3−/4−^. However, the Mo NPs@*f*-MWCNTs/SPCE exhibit a higher redox peak current and smaller peak-to-peak potential separation (Δ*E*_p_) compared to the bare SPCE and *f*-MWCNTs/SPCE. The calculated Δ*E*_p_ values for bare SPCE, *f*-MWCNTs/SPCE, and Mo NPs@*f*-MWCNTs/SPCE are shown as 130, 141 and 129 mV, respectively. The smaller anodic to cathodic peak potential separation with the excellent redox peak current of Mo NPs@*f*-MWCNTs/SPCE indicates the existence of fast electron transfer in the system. In addition, redox peak current ratio (*I*_pa_/*I*_pc_) of Mo NPs@*f*-MWCNTs/SPCE is 1.07 (1≈ reversible) which is confirmed that the reversible redox reaction of ferricyanide at Mo NPs@*f-*MWCNTs/SPCE. To find out the effective electrochemical active surface area of the electrode, the redox properties of Mo NPs@*f*-MWCNTs/SPCE was studied in [Fe(CN)6]^3−/4−^ at different scan rates (20 to 300 mVs^−1^) and shown in Fig. 5C. The electrochemically active surface area was determined from Randles–Sevcik equation,

1 $$I{\rm{p}}=(2.69\times {10}^{5})\,{{\rm{n}}}^{3/2}{{\rm{ACD}}}^{1/2}{\upsilon }^{1/2}$$

From the slopes of the *I*_pa_ vs **(**Scan rate)^1/2^(vs^−1^)^1/2^ (Fig. 5D), the electroactive surface area of the bare SPCE, *f*-MWCNTs/SPCE and Mo NPs@*f*-MWCNTs/SPCE were calculated to be 0.097, 0.184 and 0.196 cm^2^, respectively. These results indicated that the Mo NPs@*f*-MWCNTs/SPCE is a suitable electrode for effective electrochemical sensing of DA.

### Electrochemical detection of DA

**Figure 6:**
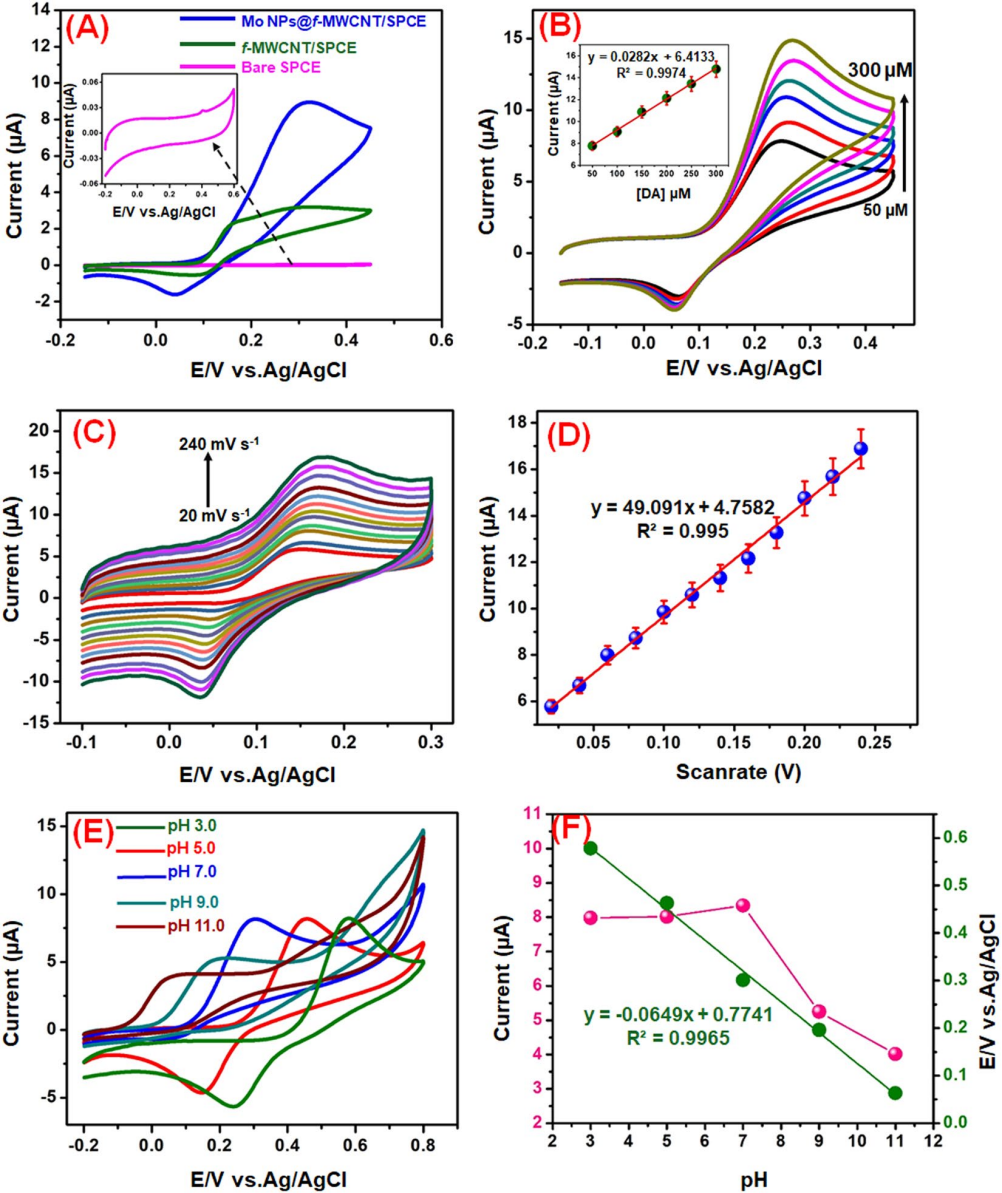
Cyclic voltammograms obtained at Bare SPCE, *f*-MWCNTs, and Mo NPs@*f*-MWCNTs nanocomposite films modified SPCE in PB (pH 7.0) containing 100 µM DA; scan rate of 50 mV s^−1^ (**A**). CVs obtained Mo NPs@*f*-MWCNTs modified electrode containing different concentrations of DA (50–300 µM) in PB (pH 7.0) at a scan rate of 50 mV s^−1^, Insert; Calibration plot of *I*_pa_ vs. conc. [DA]/µM (**B**). CV response for different scan rates from 20–240 mV s^−1^ using Mo NPs@*f*-MWCNTs modified electrode containing 100 μM DA in N_2_ saturated 0.1 M PB (**C**). Linear plot for scan rate (V) vs. *I*_pa_ of DA (**D**). Cyclic voltammograms obtained at Mo NPs@*f*-MWCNTs in supporting electrolyte of different pH contains 100 µM DA (**E**). The plot between *E*_pa_ (V) vs. pH and *I*_pa_ (µA) vs. pH (**F**).

Cyclic voltammetry (CV) technique was applied to evaluate the performance of electrochemical oxidation of DA over the bare SPCE, *f*-MWCNTs/SPCE, and Mo NPs@*f*-MWCNTs/SPCE in 0.1 M PB (7.0) containing 100 µM DA at a scan rate of 50 mV s^−1^ (Fig. 6A). Notice that, the bare SPCE did not show any obvious response towards DA. while, the *f*-MWCNTs modified electrode shows a small redox peak current towards DA, which indicates the poor electrocatalytic activity of *f*-MWCNTs. When compared with the bare SPCE and *f*-MWCNTs modified SPCE, Mo NPs@*f*-MWCNTs modified SPCE enhanced the DA oxidation and reduction currents with good reversibility, and the obtained current response was found to be 15 folds higher than that of bare SPCE. It can reveal that the negatively charged carboxyl groups in the Mo NPs@*f*-MWCNTs electrostatically interact with the amine group of DA, which significant interaction enhanced the specificity of DA. The interaction and electron transportation between Mo NPs@*f*-MWCNTs and DA further strengthened by π- π stacking forces. Also, the excellent synergistic effects that appeared between the Mo NPs and *f*-MWCNTs improve the well-conductive area and promote the electron transfer rate between the DA and the electrode surface. The electrochemical mechanism suggests that dopamine is directly oxidized into dopamine-o-Quinone (DOQ) with an equal number of protons and electrons.

The effects of different concentration of DA over the Mo NPs@*f*-MWCNTs electrode was carried out by varying the concentration of DA (50–300 µM) in 0.1 M PB (7.0), at a scan rate of 50 mV s^−1^ (Fig. 6B). The anodic peak current was enhanced linearly as the concentration of DA increased. The result also reveals that the Mo NPs@*f*-MWCNTs hybrid material can be a suitable material for the quantification of DA.

The electrochemical mechanism between the DA and Mo NPs@*f*-MWCNTs modified electrode investigated from cyclic voltammogram of 100 μM DA with different scan rate (20–240 mVs^−1^) (Fig. 6C). Figure 6D displays the linear plot between the oxidation peak currents (*I*_pa_) of DA and the scan rates. As it showed that, the linearity was observed for the *I*_pa_ of DA against scan rates. Furthermore, *I*_pa_ of DA increased with the increasing of the scan rate, while the oxidation peak potential (*E*_pa_) was shifted towards the positive side and the reduction peak potential (*E*_pc_) was shifted towards the negative side with the increase of scan rate. The linear regression equation was expressed as *I*_pa_ (μA) = 0.0282 υ (Vs^−1^) + 6.4133 (*R*^2^ = 0.997). From this scan rate study, it was understood that the electrochemical reaction of DA at core-shell Mo NPs@*f*-MWCNTs electrode was a surface controlled process.

The effect of pH (pH 3.0–11.0) on the response of 100 μM DA in the PB (7.0) at Mo NPs@*f*-MWCNTs/SPCE was investigated by CV (Fig. 6E). The relationship of pH to the *I*_pa_ and *E*_pa_ of DA were plotted and displayed in Fig. 6F. The *E*_pa_ of the DA negatively shifted when increasing the pH (3.0 to 11.0). which indicating the direct involvement of the proton in the electrochemical reaction. The regression equation for DA was described as: *E*_pa_ = −0.0649 pH + 0.7741 (*R²* = 0.996). The slop value of the equation close to the theoretical value of 59 mV pH^−1^. In this equation, D_*E*p_/D_pH_ = 2.303 mRT/nF, where m represents the number of protons and n indicates the number of electrons involved in the reaction. Which value indicating that the electrochemical oxidation of DA at core-shell Mo NPs@*f*-MWCNTs/SPCE involved two electrons and two protons. Moreover, the redox peak current of DA increased when increasing pH 3.0 to 7.0 and peak current decreased further increasing the pH 7.0 to 11.0. Therefore, pH 7.0 was fixed as the suitable pH medium for the electrochemical sensing of DA.

### Amperometric determination of DA

**Figure 7:**
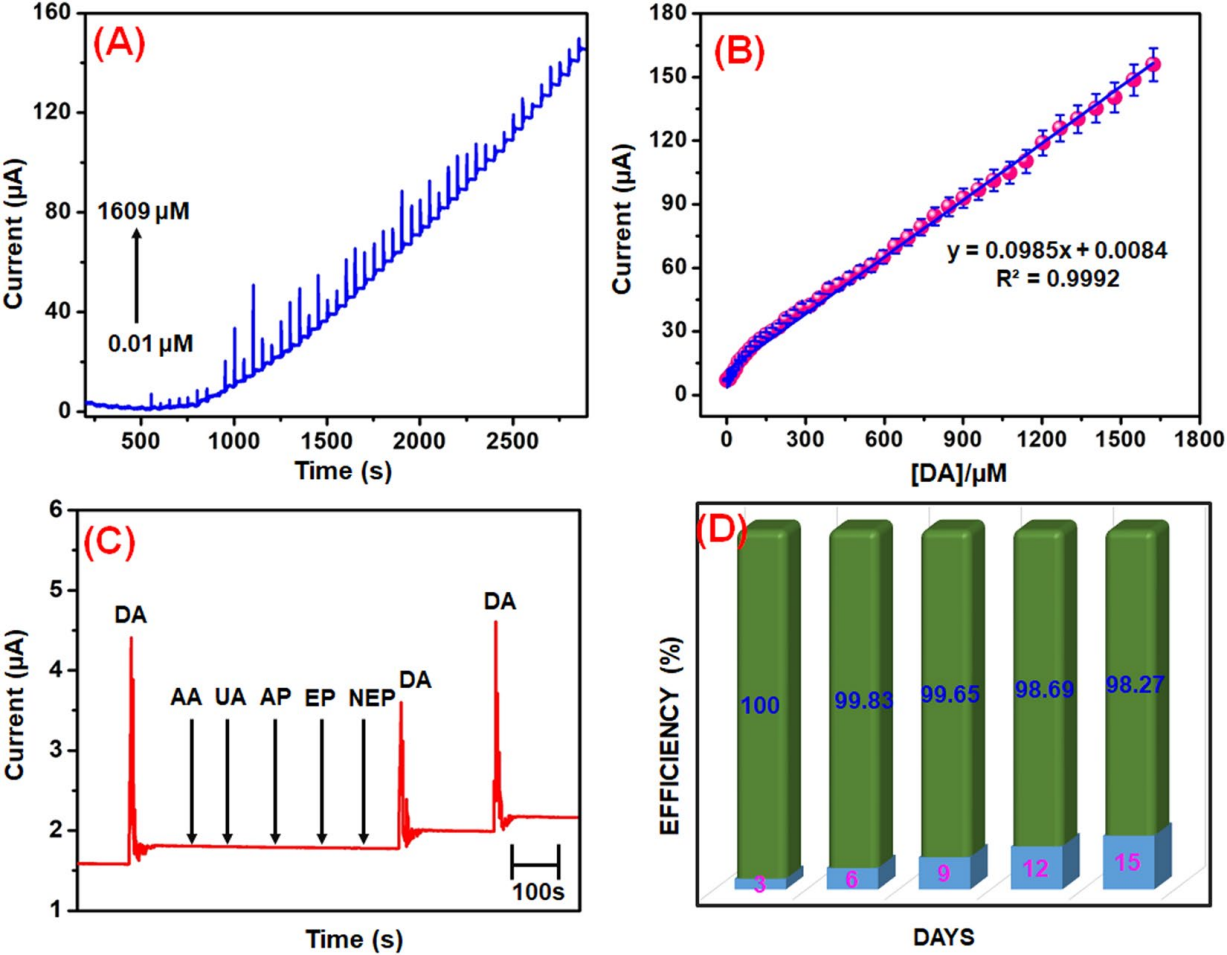
Amperometric (i–t) response at core-shell Mo NPs@*f*-MWCNTs modified RDGCE for the addition of different concentrations (0.05 to 1609 μM) of DA into the frequently stirred 0.1 M PB (pH 7.0); applied potential = 0.27 V; rotation speed = 1200 rpm (**A**). A linear plot for concentrations of DA vs. oxidation current response (**B**). At the same working conditions, amperometric response at Mo NPs@*f*-MWCNTs modified RDGCE for 50 μM of DA, 50-fold excess concentration of biologically co-interfering substances ascorbic acid (AA), uric acid (UA), acetaminophen (AP), epinephrine (EP) and norepinephrine (NEP) into continuously stirred 0.1 M PB (7.0) (**C**). The CV responses of Mo NPs@*f*-MWCNTs modified electrode towards 50 μM DA in 0.1 M PB (pH 7.0), monitored for 15 days (**D**).

The amperometric (i-t) technique is higher sensitivity and better resolution technique as compared to conventional other analytical techniques. Therefore, the amperometric technique was used for the detection of DA. Figure 7A shows the response for core-shell Mo NPs@*f*-MWCNTs modified electrode towards each continuous addition of DA (0.01–1609 µM). Every addition of DA injected at the time intervals of 50 s into the continuously stirred 0.1 PB (7.0) under the rotation speed at 1200 rpm. The applied constant working potential of the electrode was held at +0.27 V. The Mo NPs@*f*-MWCNTs modified electrode shows a well-defined and rapid amperometric current responses were obtained in various addition of DA concentration with the response time of 50 s, which is clearly indicating that the fast electron transfer process occurred between electrode and the electrolyte interface during the addition of DA. The peak currents were increased linearly with increasing the concentration of DA from lower to higher. A calibration plot was plotted for the [DA] vs. *I*_pa_ (Fig. 7B) and the linear regression equation can be expressed as *I*_pa_ (µA) = 0.0985 [DA (µM)] + 0.0084. The amperometric current responses were increased linearly with increasing the DA concentration in the linear range from 0.01 to 1609 µM. From the calibration plot, the correlation coefficient is calculated to be *R*^2^ = 0.999, and the limit of detection is estimated to be 1.26 nM according to the IUPAC definitions^47^.

2 $${\rm{LOD}}=3{\rm{\sigma }}/{\rm{q}}$$

where ‘q’ is the slope value (0.0985 µA µM^−1^) from the calibration plot, and ‘σ’ is the standard deviation obtained from the 3 measurements of the blank signal (i.e., 0.0000414 µA). the sensitivity was calculated as 4.925 µAµM^−1^ cm^−2^, respectively. The obtained analytical parameters such as linear range and LOD at core-shell Mo NPs@*f*-MWCNTs modified RDGCE towards the determination of DA have been compared with earlier reported literature and are summarized in Table 1. From the literature results, suggesting that the proposed Mo NPs@*f*-MWCNTs sensor exhibited good comparable analytical parameters with other reported electrochemical DA biosensor. This should be attributed to the excellent electrocatalytic activity of the core-shell Mo NPs@*f*-MWCNTs modified RDGCE for the sensitive and selective electrochemical determination of DA.

**Table 1:** Comparisons of the electroanalytical parameter such as a limit of detection and linear range of DA obtained at proposed core-shell Mo NPs@*f*-MWCNTs sensor with previously reported DA sensor.

Electrode	LOD (µM)	Linear range (µM)	Method	Ref.
^a^Ag_2_Se/^b^MoSe_2_/^c^GCE	0.009	0.05–1100	AMP	^3^
Graphene-^d^MoS_2_/GCE	0.007	0.05–10	CAMP	^9^
^e^MoOx/^f^SPCE	0.043	0.01–650	SWV	^5^
^g^CQD-SPCE	0.099	1–7	CV	^2^
^h^Pd@Au/^i^N,S-MGA-5	0.00036	0.001–100	DPV	^48^
^j^GR/^k^GLN	0.0045	0.05–79.5	DPV	^11^
^l^MIPs/^m^ZNTs/^n^FTO/GCE	0.02	510–800	DPV	^49^
Au-Cu_2_O/°rGO	3.9	10–90	DPV	^50^
β-MnO_2_ nanorice	0.0082	0.03–65	CAMP	^20^
Mo NPs@*f*-MWCNTs	0.00126	0.01–1609	AMP	This work

^a^Ag_2_Se; Silver selenide: ^b^MoSe_2_; molybdenum selenide: ^c^GCE glassy carbon electrode, ^d^MoS_2_; Molybdenum disulfide: ^e^MoOx; Molybdenum oxide nanoparticles: ^f^SPCE; Screen printed carbon electrode: ^g^CQD; Carbon quantum dots: ^h^Pd@Au; palladium@gold nanoalloys: ^i^N,S-MGA-5; Nitrogen and Sulphur functionalized multiple graphene aerogel: ^j^GR; graphite: ^k^GLN; gelatin: ^l^MIPs; Molecularly imprinted polymers arrays: ^m^ZNTs; ZnO nanotubes: ^n^FTO; Fluorine Tin oxide; °rGO; Reduced graphene oxide.

### Selectivity study

In order to investigate the selectivity of the core-shell Mo NPs@*f*-MWCNTs modified electrode towards the detection of DA in the presence of various possibly interfering substances by amperometric technique. As shown in Fig. 7C, the Mo NPs@*f*-MWCNTs shows well-defined amperometric current responses for the 50 μM DA, at the same time, there is no notable peak current was detected for the 50-fold excess concentration of biologically co-interfering substances ascorbic acid (AA), uric acid (UA), acetaminophen (AP), epinephrine (EP) and norepinephrine (NEP) in a continuously stirred 0.1 M PB (7.0). Also, the same amperometric signal detected for the 50 μM DA addition in the presence of aforementioned interfering substances, suggesting that the proposed core-shell Mo NPs@*f*-MWCNTs biosensor possessed excellent selectivity. Therefore, it can serve as a selective electrochemical biosensor for the detection of DA even in the existence of more interfering substances.

### Stability, reproducibility, and repeatability of the Mo NPs@*f*-MWCNTs sensor

Long-term stability of Mo NPs@*f*-MWCNTs modified electrode was investigated and stored in a PB (7.0) at room temperature. The DA signal monitored for each day by using the same Mo NPs@*f*-MWCNTs modified electrode. After 15 days, the CV response of the same Mo NPs@*f*-MWCNTs electrode has retained about 98.27% from the initial current response (Fig. 7D). This results revealed that the Mo NPs@*f*-MWCNTs modified electrode has good storage stability. The reproducibility of the modified electrode was evaluated in 0.1 M PB (pH 7.0) containing 50 μM DA at 5 independent modified electrodes with the relative standard deviation (RSD) was 2.8%, which result suggesting that acceptable reproducibility of the biosensor. The repeatability of the Mo NPs@*f*-MWCNTs sensor was studied by 5 consecutive measurements of 50 μM DA with RSD of 3.2%, indicating good repeatability of the sensor.

### Application of the non-enzymatic biosensor in real samples

The real-time application of the Mo NPs@*f*-MWCNTs biosensor was demonstrated in biological samples (human serum and rat brain) and pharmaceutical sample (Dopamine hydrochloride injection). First, rat brain sample was directly analyzed using Mo NPs@*f*-MWCNTs electrode and results were free of DA. About 0 μ, 50 μL, 100 μL, 150 μL volumes of rat brain samples were injected into 0.1 PB (7.0). Next, known concentrations of DA were injected into the rat brain sample and amperometry signals were recorded for the spiked DA sample. The Mo NPs@*f*-MWCNTs modified electrode showed well defined current responses for DA present in spiked rat brain sample. The real sample analysis was also carried out in dopamine hydrochloride injection by injecting dopamine hydrochloride injection sample into PB (pH 7.0). The capacity of the electrochemical cell was kept at 1 mL. The amperometry response recorded for each addition of DA injection sample. The direct DA detection in DA injection sample revealed the great potential of Mo NPs@*f*-MWCNTs for practical application in pharmaceutical sampling tests. In order to perform real sample analysis in a human serum sample, a known concentration of DA was spiked into serum sample. The total volume of the electrochemical cell is kept at 1 mL. Subsequently, amperometry response recorded using Mo NPs@*f*-MWCNTs modified electrode in PB containing spiked DA serum samples of different concentrations. The current response is linear with the concentration of DA in the spiked sample and hence the developed Mo NPs@*f*-MWCNTs modified electrode can be used for DA sensing in the human serum sample. Moreover, found and recovery values were calculated for specific concentrations of the spiked real samples and given as Table 2. It can be seen from the table that the core-shell Mo NPs@*f*-MWCNTs biosensor able to detects DA with an acceptable range of recoveries.

**Table 2:** Determination of DA in human blood serum, rat brain samples and dopamine hydrochloride injection using Mo NPs@*f*-MWCNTs modified electrode.

Samples	Added (µM)	Found (µM)	Recovery (%)	*RSD (%)
Human blood serum	0	0	—	0
	5	4.98	99.6	3.21
	10	10.14	101.4	3.32
Rat brain serum	0	0	0	0
	5	5.1	102	2.40
	10	10.34	103.4	2.30
Dopamine injection	0	0.25	—	—
	5	5.23	104.6	2.32
	10	10.7	107	2.71

*Related standard deviation (RSD) of 3 independent experiments.

## Conclusions

In this work, we have constructed an efficient and sensitive electrochemical biosensor based on the core-shell Mo NPs@*f*-MWCNT hybrid nanomaterial for the sensing of DA. Functionalized multiwalled carbon nanotubes (MWCNT) were entrapped by molybdenum nanoparticles (Mo NPs) to form the type of core-shell hybrid structure. The designed DA biosensor plattform with combining the advantages of MWCNT and Mo NPs offers the good electrocatalytic properties, excellent conductivity and exceptional electron transferability with fast and selective response to DA, wide linear range, low detection limit (1.26 nM, S/N = 3) and excellent selectivity. Moreover, the developed DA sensor demonstrates good reproducibility and stability. Finally, the real time application of our developed sensor was verified by the determination of DA in rat brain, human blood serum and dopamine hydrochloride injection with good accuracy. All these features validate that the core-shell Mo NPs@*f*-MWCNT hybrid nanostructure shows vast potential for the applications in electrochemical biosensing platform.
